# Diagnosis of Peripheral Pulmonary Lesions with Transbronchial Lung Cryobiopsy by Guide Sheath and Radial Endobronchial Ultrasonography: A Prospective Control Study

**DOI:** 10.1155/2021/6947037

**Published:** 2021-09-28

**Authors:** Ling Jiang, Jian Xu, Chunfang Liu, Na Gao, Jing Zhao, Xue Han, Xiaolin Zhang, Xiaohui Zhao, Yuan Liu, Weixue Wang, Junjun Zhao

**Affiliations:** ^1^Department of Respiratory and Critical Care Medicine, Dalian Municipal Central Hospital Affiliated of Dalian Medical University, Dalian 116033, China; ^2^Department of Anesthesiology Medicine, Dalian Municipal Central Hospital Affiliated of Dalian Medical University, Dalian 116033, China; ^3^Department of Pathology Medicine, Dalian Municipal Central Hospital Affiliated of Dalian Medical University, Dalian 116033, China

## Abstract

**Objective:**

We design a prospective control study on the utilization of transbronchial cryobiopsy guided by EBUS-GS (EBUS-GS-TBCB) to diagnose PPLs.

**Methods:**

PPLs were defined as pulmonary nodules or masses with a diameter from 10 mm to 50 mm. PPLs were randomly divided into group EBUS-GS-TBCB and transbronchial biopsy by forceps guided under EBUS-GS (EBUS-GS-TBB).

**Results:**

28 cases were involved in group EBUS-GS-TBCB and 31 cases were in group EBUS-GS-TBB. The mean sizes of PPLs were 30.23 ± 11.10 mm in group EBUS-GS-TBCB and 28.69 ± 8.62 mm in group EBUS-GS-TBB (*t* = 0.600, *p*=0.551). The diagnostic yields of EBUS-GS-TBCB and EBUS-GS-TBB were 75% and 64.52% respectively, and the difference between the two groups was not significant (*χ*^2^ value = 0.137, *p*=0.711). If only the first specimen was taken into account, the diagnostic yields from EBUS-GS-TBCB and EBUS-GS-TBB were 64.29% (18/28 cases) and 35.48% (11/31 cases), respectively. The difference was statistically significant by Fisher's Exact Test (*χ*^2^ value = 4.883, *p*=0.038). The total incidence rates of bleeding were 21.43% and 6.45%, respectively, in groups EBUS-GS-TBCB and EBUS-GS-TBB. The total incidence rates of pneumothorax were 7.14% and 0, respectively, in groups EBUS-GS-TBCB and EBUS-GS-TBB.

**Conclusion:**

The diagnostic yield of EBUS-GS-TBCB was slightly higher than that of EBUS-GS-TBB for the diagnosis of PPLs. EBUS-GS-TBCB might be useful if only the first sample was taken into account.

## 1. Introduction

Pulmonary nodules are becoming an increasingly common radiographic finding. The probability of malignancy has to be evaluated according to the management of pulmonary nodules [1, 2]. The malignant evidence is based on both clinical and radiographic features [3]. Some noncalcified nodules with features of mixed ground glass opacity, pleural retraction, lobular sign, and spiculation were suspicious for lung cancer. The growth on serial imaging is highly suggestive of malignancy. Rapid identification of these nodules with the likelihood of malignancy is critical in improving the survival of lung cancer and is worthy of all efforts. The pathological diagnosis before surgical excision may avoid futile invasive procedures for benign nodules. When biopsy is recommended, transthoracic needle aspiration or core biopsy (TTCB) is currently preferred because it has a fairly high diagnostic yield regardless of pneumothorax and hemorrhage. Flexible bronchoscopy is another diagnostic option. But, the sensitivity of conventional transbronchial biopsy is depending on the size and location of the nodules. Common bronchoscopy is difficult to visualize peripheral pulmonary nodules sited at a labyrinth of subsegmental bronchi and branches if without guidance. Many advances on interventional diagnostic bronchoscopy have emerged which offer guidance to cross the bronchial tree to reach nodules [4]. Endobronchial ultrasonography with a guide sheath (EBUS-GS) is a kind of technology that combines bronchoscopy with radial endobronchial ultrasound (R-EBUS); the transbronchial biopsy by forceps (TBB) can be introduced by the guide sheath (GS) which lead to lesions. The biopsy combined with EBUS-GS had a significantly higher diagnostic yield than other guidance alone [5]. Recently, flexible cryoprobes have been used for peripheral lung parenchymal biopsy. Studies on transbronchial cryobiopsy (TBCB) have shown the improvements in diagnostic yield, sample size, and architectural preservation of the biopsy specimens to diagnose diffuse parenchymal lung diseases [6–8]. We design a prospective control study on the utilization of TBCB guided by EBUS-GS (EBUS-GS-TBCB) to diagnose peripheral pulmonary lesions (PPLs). The diagnostic yield and safety of EBUS-GS-TBCB are observed by comparing with those of EBUS-GS-TBB in this research.

## 2. Methods

**Figure 1:**
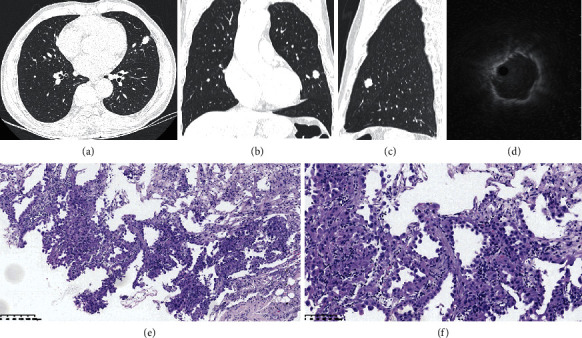
A peripheral pulmonary lesions (PPLs) with high malignant likelihood. The bronchus sign, pleural retraction, lobular, and spiculation were displayed on chest CT based on axial (a), coronal (b), and sagittal (c) images. The eccentric image of R-EBUS around target bronchus inferred that cryoprobe was within PPLs (d). A biopsy was performed by EBUS-GS-TBCB and adenocarcinoma was diagnosed on pulmonary pathological slide (HE stained ×100 (e) and ×200 (f) magnification times).

We performed a prospective study at a tertiary care academic medical center. Subjects with PPLs detected by chest CT or HRCT in a recent month were collected at the Department of Respiratory and Critical Care Medicine in Dalian Municipal Central Hospital affiliated to Dalian Medical University from March 2019 to June 2020. PPLs were defined as pulmonary nodules or masses with a single, well-circumscribed boundary surrounded by pulmonary parenchyma in which the sizes were measured from 10 mm to 50 mm in diameter. The single dominant nodule or mass accompanied by one or more incidental small nodules was also involved if the dominant lesions were 10 mm to 50 mm in diameter. The enrolment criteria were as follows: (a) the ranges of age were from 18 to 75 years; (b) PPLs with a likelihood of malignancy were solid or part-solid in which solid components were more than 50%; and (c) the bronchus signs on the heart side of PPLs were displayed on chest CT or HRCT (Figure 1). The exclusion criteria were as follows: (a) contraindications for bronchoscopy; (b) PPLs with an endobronchial component observed by common bronchoscopy; and (c) PPLs associated atelectasis or obstructive pneumonia. The size of the PPLs and the shortest pleural distance from the center of lesions to adjacent pleural, involving parietal, mediastinal, and interlobar pleural, were measured on the axial lung window setting of chest CT. The largest diameter, the shortest pleural distance, and the location of PPLs were recorded. PPLs with the size of 10 mm to 20 mm at superior lung areas had to be allocated according to the ratio of 1 : 1 and others were randomly divided into groups EBUS-GS-TBCB and EBUS-GS-TBB with single blind method. Superior lung areas included apical, anterior, and posterior pulmonary segments of bilateral upper lung lobes. Endobronchial ultrasound-guided transbronchial needle aspiration biopsy (EBUS-TBNA) was simultaneously conducted in those with the enlarged mediastinal or hilar lymph nodes.

This study followed ethics principles for medical research in the Declaration of Helsinki and was approved by Dalian Municipal Central Hospital Human Ethics Committee (2019-004-10). Written informed consent was obtained from all subjects or their guardians before the examination.

Blood clotting function, arterial blood gas, electrocardiography, and cardiac and pulmonary function were evaluated before bronchoscopy. The examination was prohibited in patients with a forced vital capacity of less than 50%, respiratory failure, or cardiac insufficiency. Some anticoagulation agents, such as aspirin, clopidogrel, and warfarin, had to be blocked for five days and be replaced with low molecular heparin. Solids and liquids were withheld before 6 hours. The target pulmonary subsegment in which PPLs located should be confirmed in advance depending on chest CT or HRCT.

All examinations were performed by bronchoscopy (Olympus, BF-1T260, Japan). Subjects accepted intravenous remifentanil and disoprofol anesthesia and breathed through mechanical ventilation through a size 4 laryngeal mask airway. The examination of bronchoscopy had to be terminated when severe bleeding appeared and a blocking balloon would be inserted into the target segmental bronchus from the working canal.

EBUS-GS-TBB was performed by a guiding sheath (Olympus, Single Use Guide Sheath Kit K-203 with 2.6 mm external diameter, Japan) under the guidance of R-EBUS (Olympus, UM-S20-17S with 1.7 mm diameter, Japan) according to the procedures in [9, 10]. The sheath-covered R-EBUS probe was inserted into the working channel and searched each branch of target subsegment to find out typical images of PPLs. Once a typical R-EBUS image identified with PPLs was obtained, the probe was withdrawn while GS was left in place. Biopsies by forceps and brush (Olympus, Single Use Guide Sheath Kit K-203, Japan) were introduced via GS to provide specimens for pathological and cytological examination. Biopsies were repeated until 10 pieces of specimens were sampled unless the tissues were not enough.

EBUS-GS-TBCB was performed by a cryoprobe (ERBE, ERBOKRYO CA with 1.9 mm diameter, Germany) through a guiding sheath (specially designed with 2.1 mm internal diameter, 2.6 mm external diameter, and 85 mm length, China) under the guidance of R-EBUS (Olympus, UM-S20-17S with 1.7 mm diameter, Japan). The metal head of the cryoprobe must jut completely out of the far end of the sheath and the length was equal to extending part of the ultrasonic probe from the sheath. The total length of extending part of the ultrasonic probe and the sheath should be measured in advance and the equal length on the cryoprobe should be marked. The procedures to search PPLs and indwell GS were the same as EBUS-GS-TBB. Once a typical EBUS image identified with PPLs was obtained, the probe was withdrawn while the GS was left in place. The cryoprobe was inserted into the GS to the marked level and cooled for 4 to 6 seconds at the desired location under 50-bar pressure of CO_2_. Then, the cryoprobe was firmly pulled out with GS and bronchoscopy. The frozen samples on the cryoprobe were separated from PPLs. The cool duration should be computed before each examination depending on the time when the cryoprobe was frozen in a basin. Three pieces of specimens were usually sampled in each subject unless GS could not be placed in the same position again or bleeding hampered biopsy.

The positive R-EBUS detection was typical concentric or eccentric images that defined the location of probe within PPLs [11]. The concentric image was that the probe was completely surrounded by the lesion and the eccentric image (Figure 1) was that the probe was within but largely biased toward one side at the edge of the lesion. The adjacent image that the probe was not within the lesion [12] or other images were recorded as the failure of detection by R-EBUS. The procedures of bronchoscopy were recorded as the failure of insertion if the ultrasonic probe with guide sheaths could not be placed into the target pulmonary segment bronchus. The subjects that failed to detect or insert had to quit the group due to the absent pathological diagnosis. EBUS-GS-TBB was conducted subsequently by a thin sheath (Olympus, Single Use Guide Sheath Kit K-201, Japan) and thin bronchoscopy (Olympus, BF-6C260 with 4.8 mm external diameter and 2.0 mm working channel, Japan). VBN and fluoroscopy were performed to guide thin bronchoscopy.

All patients were kept horizontal position without pillow, inhaled oxygen, supervised blood pressure and pulse oxygen for 4 hours after examination. X-ray film should be taken 24 hours later for evaluating pneumothorax.

All complications were observed during and after examinations. The bleeding was evaluated as light, severe, and fatal degree. Severe bleeding required intravenous hemostatic drug administration, cessation of transbronchial procedure, and inflation of the bronchial blocking balloon. Fatal bleeding was associated with cardiopulmonary instability, transfusion of packed RBCs, or surgical intervention. Pneumothorax was evaluated as light, severe, and fatal degrees. Light means the lung volume was collapsed by less than 50% and severe means pulmonary volume was collapsed, combined with mediastinal emphysema or gas drainage of the pleural cavity. Fatal pneumothorax was associated with surgical intervention. Other complications were also evaluated such as subcutaneous emphysema, vocal cord lesions, and infection of the bronchus and lung.

Malignant PPLs were diagnosed by pathology of transbronchial biopsy, EBUS-TBNA, TTCB guided by chest CT, or surgical resection. Benign PPLs needed to be confirmed by either another pathology or follow-up for at least 6 months until the lesions improved radiographically. The stable or growing benign PPLs were defined as unimproved and surgical resection was recommended because of the rising malignant likelihood. Diagnostic yield and incidence rate of the complications were calculated in groups EBUS-GS-TBCB and EBUS-GS-TBB.

### 2.1. Statistical Analysis

The data were analyzed by using SPSS (IBM Corporation, New York, USA). The measurement data were reported as the mean values ± standard deviations. Count data were analyzed using Pearson Chi-Square tests. The diagnostic sensitivity, specificity, and accuracy were calculated. The diagnostic sensitivity was defined as the probability of true positive subjects in all suffering the same diagnosis. The specificity was defined as the rate of true negative subjects in all nonsuffering diseases. *p* values were double-sided, and *p* < 0.05 meant that differences were significant.

## 3. Results

### 3.1. Participant

**Figure 2:**
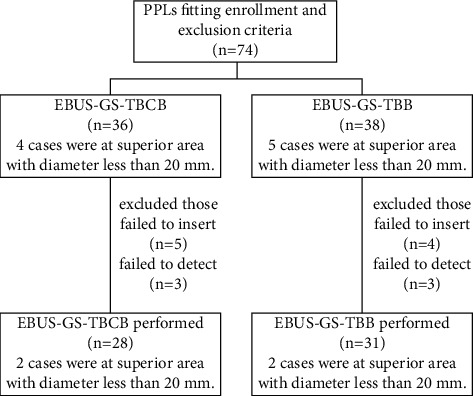
A diagram showing how the study population was divided into groups for analysis (*n* = 74). EBUS-GS-TBCB: endobronchial ultrasonography with a guide sheath-guided transbronchial cryobiopsy; EBUS-GS-TBB: endobronchial ultrasonography with a guide sheath-guided transbronchial biopsy by forceps.

74 subjects were enrolled and 15 subjects withdrew because of failing to detect PPLs by R-EBUS or failing to insert the guide sheaths (Figure 2). Finally, 59 subjects remained because biopsies by EBUS-GS-TBB or EBUS-GS-TBCB were completed (data had been shared in the supplement files). The clinical data of the remaining subjects are shown in Table 1. The total rate of failing insertion was 12.16% (9/74 cases). The failure of insertion was 13.89% (5/36 cases) and 10.52% (4/38 cases), respectively, in groups EBUS-GS-TBCB and EBUS-GS-TBB (*χ*^2^ value = 0.196, *p*=0.658). The failure of insertion appeared only in superior lung areas in both groups, especially for lesions at areas affiliated with apical pulmonary segments. The total rate of failing to detect by R-EBUS was 8.10% (6/74 cases). The failure of detection was 8.33% (3/36 cases) and 7.89% (3/38 cases), respectively, in groups EBUS-GS-TBCB and EBUS-GS-TBB (*χ*^2^ value = 0.005, *p*=0.945). The failure of detection appeared equally at superior, middle, and inferior lung areas. All subjects were followed up for more than 6 months and the longest follow-up time was 12 months.

### 3.2. Diagnostic Yield

The subjects diagnosed by pathology and follow-up are displayed in Table 2. Surgical resection was performed in 23 subjects after bronchoscopy, endobronchial ultrasound-guided transbronchial needle aspiration biopsy (EBUS-TBNA) was performed simultaneously in 11 subjects, TTCB guided by chest CT was performed in 5 cases because of inflammation lesions by transbronchial pathology, and all subjects were followed up to supervise therapeutic effects. In group EBUS-GS-TBCB, 17 subjects got 3 biopsied samples, 9 subjects got 2 samples, and 2 got only 1 sample because of severe bleeding. The mean number of passes in cryobiopsy was 3.1 ± 1.1 times. The mean activation time of cryobiopsy was 4.7 ± 0.8 seconds, and the median time was 5 seconds. In group EBUS-GS-TBB, 27 subjects got 10 biopsied samples and 4 subjects got less than 10 samples.

As for inflammatory PPLs diagnosed by initial pathology in group EBUS-GS-TBCB, 6 cases were confirmed as malignancy by subsequent other pathology, 2 improved cases were confirmed as pneumonia and 1 stable case did not confirm the diagnosis owing to refusing biopsy again or surgical resection (Table 2). In group EBUS-GS-TBB, 7 were confirmed as malignancy by other biopsies or surgical resections, 3 improved PPLs were confirmed as pneumonia depending on radiological following, 2 were confirmed inflammation by TTCB guided by chest CT, and 4 unimproved PPLs did not confirm the diagnosis. Three malignant subjects could not get pathological type before surgical resection because specimens were not enough from EBUS-GS-TBB (Table 2). Two subjects got only one biopsy owing to severe bleeding in group EBUS-GS-TBCB, adenocarcinoma was diagnosed by initial pathology in one case, and inflammation was confirmed in another one by follow-up. All above information of diagnostic tests is mentioned in Supplement Table 1.

Finally, the diagnostic yields of EBUS-GS-TBCB and EBUS-GS-TBB were 75% (21/28 cases) and 64.52% (20/31 cases), respectively. The diagnostic yield in group EBUS-GS-TBCB seemed to be slightly higher. But, the difference was not significant by Pearson's chi-squares test (*χ*^2^ value = 0.137, *p*=0.711). The diagnostic sensitivity, specificity, and accuracy were 76% (19/25 cases), 25% (2/8 cases), and 77.78% (21/27 cases), respectively, in group EBUS-GS-TBCB and 68.18% (15/22 cases), 41.67% (5/12 cases), and 74.07% (20/27 cases), respectively, in group EBUS-GS-TBB. The values by Pearson's chi-squared test in the two groups were 0.357, 0.290, and 0.101, the differences were not significant, and *p* values were 0.550, 0.590, and 0.750, respectively. If only the first specimen was taken into account, the diagnostic yields from EBUS-GS-TBCB and EBUS-GS-TBB were 64.29% (18/28 cases) and 35.48% (11/31 cases), respectively (Table 3) and the difference was statistically significant by Fisher's Exact Test (*χ*^2^ value = 4.883, *p*=0.038).

### 3.3. The Complication

Bleeding occurred in both groups, and pneumothorax occurred only in group EBUS-GS-TBCB (Table 1). The total incidence rates of bleeding in groups EBUS-GS-TBCB and EBUS-GS-TBB were 21.43% (6/28 cases) and 6.45% (2/31 cases), respectively (*χ*^2^ value = 2.816, *p*=0.093). Pneumothorax rates were 7.14% (2/28 cases) and 0, respectively, in groups EBUS-GS-TBCB and EBUS-GS-TBB (*χ*^2^ value = 2.292, *p*=0.130). There were 5 and 3 cases, respectively, in groups EBUS-GS-TBCB and EBUS-GS-TBB where the shortest pleural distances were less than 10 mm. The distances were 8.61 mm and 36.84 mm, respectively, in two cases of pneumothorax (Supplement Table 2). Fatal bleeding and other complications did not occur.

## 4. Discussion

The advantages of traditional bronchoscopy in the detection of PPLs were little because lesions locating beyond the subsegmental bronchus were difficult to be found out. Advances on diagnostic techniques of interventional pulmonology provided the guidance to localize PPLs and improve the diagnostic yields. These advances involved R-EBUS and GS, electromagnetic navigation bronchoscope (ENB), virtual bronchoscopic navigation (VBN), thin/ultrathin bronchoscope, and so on [4, 13, 14]. The diagnostic yields of traditional bronchoscopy with guidance were significantly higher than those without guidance [5, 15]. The pooled diagnostic yield could improve to 70% by using several guiding techniques for nodules diagnosis [15]. The utility of EBUS-GS guiding the diagnosis of PPLs was the most studied. The overall weighted diagnostic yield was 70.6% in a meta-analysis [16]. The diagnostic yield of conventional TBB following the initial EBUS-GS-TBB was 48% [9]. The diagnostic yield of EBUS-GS-TBB under X-ray fluoroscopy (64.6%) was higher than TBB guided only by X-ray fluoroscopy without EBUS-GS (46.7%) [5, 17]. Although the diagnostic yield of less than 10 mm nodules was similar to those with the larger size in [10], it was varied by sizes of PPLs in most cases. The diagnostic yields of EBUS-GS-TBB were 58%, 72%, 77%, 87%, 88%, respectively, in pulmonary nodules with the diameter of 1–2 cm, 2.1–3 cm, 3.1–4 cm, 4.1–5 cm, and larger than 5.1 cm [5, 11]. The diagnostic yield was significantly higher for lesions with sizes larger than 2 cm, malignant features, or bronchus sign on CT scans [11]. The failure of R-EBUS guiding to detect PPLs was also presented in much research. Only 81.5% of lesions could be checked out by R-EBUS [17]. 4% PPLs could not be identified by using R-EBUS [5]. In our research, the failure of detection by R-EBUS was 8.10% and the diagnostic yield of EBUS-GS-TBB was 64.52%. Orientation of R-EBUS images might be an important factor affecting diagnostic yield. The diagnostic yield of TBCB in eccentrically and adjacently orientated lesions was higher than EBUS-GS-TBLB [12]. However, this diagnostic yield was not evaluated because few eccentric lesions were involved in our groups. It might be something to do with required bronchus signs in our eligible subjects. The CT-based image system was another rapidly expanded technique for guiding bronchoscope in procedure of diagnosing PPLs. VBN and ENB could plan the optimal pathways to the PPLs in advance by reconstruction of chest CT [18]. VBN alone as the guidance did not improve the diagnostic yield of PPLs [19, 20]. The diagnostic yield of the VBN-assisted group was 76.9% and was lower than 85.9% of the X-ray fluoroscopy assisted group [20]. The diagnostic yield could reach 74.3% if VBN combined EBUS-GS as the guidance and it was slightly higher than 72.3% only by EBUS-GS without VBN [5]. As for the ENB-aided samples, malignant results were obtained from 63.3% subjects [21]. The 12-month diagnostic yield was 73% when ENB combined fluoroscopy and R-EBUS guiding transbronchial biopsy in PPLs [22]. Advances on bronchoscope making allowed inserting thin/ultrathin bronchoscope into more distal bronchi within or adjacent to PPLs. The diagnostic yield of thin bronchoscope guided by R-EBUS was 49% and slightly higher than 37% in standard bronchoscope guided by X-ray fluoroscopy [23]. The diagnostic yield of ultrathin bronchoscope guided by EBUS-GS was 70.1% and significantly higher than 58.7% in thin bronchoscope [24]. The diagnostic yield of VBN assisting ultrathin bronchoscope was 67.1% and was not significantly higher than 59.9% of the non-VBN assisting group [19].

Among PPLs failing to be detected by R-EBUS, cone-beam computed tomography guiding thin bronchoscope might potentially increase both navigation and diagnostic yield [25]. Bronchoscopic trans-parenchymal nodule access could guide bronchoscope to create a straight, vessel-free, and trans-parenchymal path to nodules [26].

However, the transbronchial biopsy was always by forceps in spite of the many advances achieved in various guiding methods and thin/ultrathin bronchoscopy. TBCB might be a breakthrough on biopsy patterns. Many studies had displayed that samples by TBCB were larger than those by forceps for diagnosis of diffuse parenchymal lung diseases [6–8]. Larger samples allowed more tissues for pathological diagnosis and further molecular analyses. So, several small sample research works explored the diagnosis of PPLs by TBCB guided with EBUS-GS. Schuhmann et al. [27] designed research in 39 subjects that PPLs were biopsied either by TBCB following TBB or by TBB following TBCB guided with EBUS-GS randomly. The diagnostic yield of TBCB in PPLs visualized by EBUS was 74.2%; meanwhile, 4 cases were successfully diagnosed only by cryobiopsy [27]. Taking only the first biopsy into account, 61.3% of subjects were diagnosed by cryobiopsy [27]. Arimura et al. [28] observed TBCB following TBB guided with EBUS-GS in 23 subjects. The sensitivity, specificity, and diagnostic accuracy could reach 85%, 100%, and 87% for cryobiopsy with one or two times, and the difference was not significant with five times TBB. We designed a control study in 60 subjects that were divided into two groups and the performance of EBUS-GS-TBCB and EBUS-GS-TBB to diagnose PPLs was compared. The diagnostic yield by EBUS-GS-TBCB was 75% and similar to the research. The diagnostic yield from the first sample of EBUS-GS-TBCB was 64.29% and significantly differed from that of EBUS-GS-TBB (35.48%) in our research. Kho et al. [12] retrospective diagnostic yield of TBCB guided with EBUS-GS and fluoroscopy in 38 subjects. 41.2% (15 cases) and 15.8% (9 cases), respectively, of the lesions were eccentric and adjacent orientation depending on images of R-EBUS. TBCB could significantly increase the diagnostic yield for eccentrically and adjacently orientated lesions than TBB. Unfortunately, only 28.57% (8 cases) and 19.35% (6 cases) of lesions, respectively, were eccentric in groups EBUS-GS-TBCB and EBUS-GS-TBB in our research (Table 1); this might relate to eligible PPLs accompanying bronchus sign.

On the whole, diagnostic yield of EBUS-GS-TBCB was slightly higher than EBUS-GS-TBB for diagnosis of PPLs. EBUS-GS-TBCB might be useful if only the first sample was taken into account.

## Figures and Tables

**Table 1:** The clinic data of the final remaining subjects.

		EBUS-GS-TBCB	EBUS-GS-TBB	*p*
Final remaining cases		47.46% (28/59)	52.54% (31/59)	
Sex	Male	64.29% (18/28)	64.52% (20/31)	0.985
	Female	35.71% (10/28)	35.48% (11/31)	
Mean age (years)		64.50 ± 7.78	61.84 ± 11.67	0.313
Pulmonary comorbidities	Emphysema	14.29% (4/28)	6.45% (2/31)	0.320
	Interstitial disease	10.71% (3/28)	9.68% (3/31)	0.895
Malignant history	Never	92.86% (26/28)	96.77% (30/31)	0.494
	Ever	7.14% (2/28)	3.23% (1/31)	
Mean size of PPLs		30.23 ± 11.10 mm	28.69 ± 8.62 mm	0.551
Pleural distances		23.79 ± 13.58 mm	20.28 ± 8.30 mm	0.241
Orientation of R-EBUS	Concentric	71.43% (20/28)	80.65% (25/31)	0.985
	Eccentric	28.57% (8/28)	19.35% (6/31)	
Distribution	Superior	32.14% (9/28)	32.26% (10/31)	0.992
	Middle	17.85% (5/28)	9.68% (3/31)	0.359
	Inferior	50% (14/28)	58.06% (18/31)	0.535
Bleeding complication	Light	14.28% (4/28)	6.45% (2/31)	0.320
	Severe	7.14% (2/28)	0	0.130
	Fatal	0	0	
Pneumothorax complication	Light	3.57% (1/28)	0	0.289
	Severe	3.57% (1/28)	0	0.289

Superior, superior lung area includes apical, anterior, and posterior pulmonary segments of left and right upper lobe; middle, middle lung area includes the right middle lobe and lingual pulmonary segment of left upper lobe; inferior, inferior lung area includes left and right lower lobe.

**Table 2:** The case of diagnosis confirmed by pathology and follow-up.

	EBUS-GS-TBCB			EBUS-GS-TBB		
Adenocarcinoma	14			7		
Squamous carcinoma	1			2		
Small cell lung cancer	0			2		
Sarcomatoid carcinoma	1			0		
Metastatic carcinoma	1 (from renal carcinoma)			1 (from breast cancer)		
Pulmonary tuberculosis	2			0		
Malignancy	0			3	Adenocarcinoma	3^a^
Inflammation	9	Adenocarcinoma	3^a^	16	Adenocarcinoma	3^a^
			1^b^			1^c^
		Small cell lung cancer	1^c^		Small cell lung cancer	3^c^
		Inflammation	1^b^		Inflammation	2^b^
		Pneumonia	1^d^		Pneumonia	3^d^
		No conclusion	1^d^		No conclusion	4^d^
		Metastatic carcinoma from breast	1^b^			

a, PPLs were confirmed by surgical resection; b, PPLs was confirmed by transthoracic core biopsy guided by chest CT; c, PPLs was confirmed by endobronchial ultrasound-guided transbronchial needle aspiration biopsy; d, PPLs were confirmed by follow-up.

**Table 3:** The diagnostic yield from the first specimen.

	Positive cases	Positive rate (%)	*χ*^2^ value	*p*
EBUS-GS-TBCB	18/28	64.29	4.883	0.038
EBUS-GS-TBB	11/31	35.48		

## Data Availability

The data used to support the findings of the study are provided in the supplementary information files.

## Supplementary Materials

Supplementary Materials The clinical information and final diagnostic tests groups EBUS-GS-TBCB and EBUS-GS-TBB are shown in Supplement Table 1. The final diagnosis, the shortest pleural distance, orientation of radial image, and complication in groups EBUS-GS-TBCB and EBUS-GS-TBB are shown in Supplement Table 2.
