# Supplementary material for: Diagnosis of Peripheral Pulmonary Lesions with Transbronchial Lung Cryobiopsy by Guide Sheath and Radial Endobronchial Ultrasonography: A Prospective Control Study
Source: Can Respir J. 2021 Sep 28;2021:6947037. doi: 10.1155/2021/6947037 (PMC8492292; doi:10.1155/2021/6947037)
Supplement: Supplementary Materials — The clinical information and final diagnostic tests groups EBUS-GS-TBCB and EBUS-GS-TBB are shown in Supplement Table 1. The final diagnosis, the shortest pleural distance, orientation of radial image, and complication in groups EBUS-GS-TBCB and EBUS-GS-TBB are shown in Supplement Table 2. [file 6947037.f1.zip › 6947037.f1/supplement 1(2) (1).doc]

Supplement Table 1 the clinical information and final diagnostic tests

| Grouping | Cases | Name | Gender | Age | Position | distribution | Transbronchial pathology | Operation | TTCB | EBUS-TBNA | Follow |
| --- | --- | --- | --- | --- | --- | --- | --- | --- | --- | --- | --- |
| 1 | 655793 | Shi Enhai | 1 | 79 | L10 | 3 | sarcomatoid carcinoma |  |  |  |  |
| 1 | 657456 | Qu Jianguo | 1 | 65 | R1 | 1 | adenocarcinoma |  |  |  |  |
| 1 | 659717 | Chen Anzuo | 1 | 50 | R10 | 3 | squamous carcinoma |  |  |  |  |
| 1 | 661361 | Hou Jiaqi | 1 | 68 | L2 | 1 | adenocarcinoma | 1 |  |  |  |
| 1 | 388006 | Mu Jianguo | 1 | 63 | R9+10 | 3 | adenocarcinoma |  |  |  |  |
| 1 | 680612 | Dong Zhenfu | 1 | 66 | R1 | 1 | adenocarcinoma | 1 |  |  |  |
| 1 | 680175 | Zhang Tao | 1 | 53 | L9 | 3 | adenocarcinoma | 1 |  |  |  |
| 1 | 681610 | Chi Yunping | 2 | 60 | R3 | 1 | granuloma TB |  |  |  |  |
| 1 | 693890 | Sun Qingrong | 2 | 74 | R3 | 1 | adenocarcinoma | 1 |  |  |  |
| 1 | 157386 | Wu Yanfeng | 2 | 64 | R8 | 3 | granuloma TB |  |  |  |  |
| 1 | 681468 | Li Wenlai | 1 | 61 | R8 | 3 | Inflammation |  |  | SCLC |  |
| 1 | 658457 | Liu Guoliang | 1 | 65 | R4 | 2 | kidney cancer metastasis |  |  |  |  |
| 1 | 669540 | Wang Xiaoyuan | 2 | 51 | L9 | 3 | adenocarcinoma |  |  |  |  |
| 1 | 671001 | Chao Gonghe | 1 | 74 | L3 | 1 | inflammation | adenocarcinoma |  |  |  |
| 1 | 673289 | Hou Liping | 2 | 66 | R2+3 | 1 | adenocarcinoma |  |  |  |  |
| 1 | 698751 | Wang Peiyuan | 1 | 45 | L3 | 1 | inflammation | adenocarcinoma |  |  |  |
| 1 | 698749 | Cui Weiqing | 1 | 66 | R5 | 2 | inflammation |  | adenocarcinoma |  |  |
| 1 | 180188 | Yang Xiyou | 1 | 66 | L10 | 3 | inflammation | adenocarcinoma |  |  |  |
| 1 | 600642 | Jiang Dejun | 1 | 72 | R10 | 3 | inflammation |  | inflammation |  |  |
| 1 | 699401 | Shi Shufa | 1 | 72 | R8 | 3 | adenocarcinoma | 1 |  |  |  |
| 1 | 699927 | Wang Mingchun | 1 | 74 | L2 | 1 | adenocarcinoma | 1 |  |  |  |
| 1 | 554767 | Zhang Zongxian | 1 | 67 | R4 | 2 | adenocarcinoma | 1 |  |  |  |
| 1 | 187362 | Liu Huanyu | 2 | 69 | R5 | 2 | inflammation |  | Breast cancer metastasis |  |  |
| 1 | 627563 | Yu Guilan | 2 | 68 | L7 | 3 | adenocarcinoma |  |  | 1 |  |
| 1 | 704242 | Wang Hongwei | 2 | 65 | R9 | 3 | Inflammation | granuloma |  |  | no conclision |
| 1 | 705628 | Han Min | 2 | 61 | L8 | 3 | adenocarcinoma |  |  |  |  |
| 1 | 705489 | Yao Shuhua | 2 | 62 | R9 | 3 | Inflammation | a |  |  | hylactic pneumoni |
| 1 | 566434 | Li Xueming | 1 | 60 | L5 | 2 | adenocarcinoma | 1 |  |  |  |
| 2 | 643684 | Sun Maosong | 1 | 56 | L9 | 3 | SCLC |  |  |  |  |
| 2 | 651563 | Gao Shenglin | 1 | 74 | R2+3 | 1 | adenocarcinoma |  |  |  |  |
| 2 | 653893 | Wen Guichun | 2 | 64 | L9 | 3 | adenocarcinoma | 1 |  |  |  |
| 2 | 653638 | Zhang Wei | 1 | 61 | L2 | 1 | inflammation | adenocarcinoma |  |  |  |
| 2 | 656123 | Zhang Chunyun | 2 | 66 | R1 | 1 | carcinoma | adenocarcinoma |  |  |  |
| 2 | 656657 | Jia Xuemei | 2 | 50 | R10 | 3 | inflammation |  |  |  | no conclision |
| 2 | 658493 | Jiang Chengshuang | 1 | 63 | R5 | 2 | adenocarcinoma | 1 |  |  |  |
| 2 | 658516 | Zhang Yicheng | 1 | 55 | R4 | 2 | SCLC |  |  |  |  |
| 2 | 663861 | Wang Xiaoxiao | 2 | 32 | R6 | 3 | malignancy | adenocarcinoma |  |  |  |
| 2 | 666416 | Li Zhigeng | 1 | 67 | L10 | 3 | squamous carcinoma |  |  |  |  |
| 2 | 676813 | Shi Baosheng | 1 | 79 | L4 | 2 | malignancy | adenocarcinoma |  |  |  |
| 2 | 679360 | Lu Mengjing | 1 | 60 | L8 | 3 | adenocarcinoma | 1 |  |  |  |
| 2 | 684333 | Wang Baozhen | 1 | 79 | R6 | 3 | inflammation | adenocarcinoma |  |  |  |
| 2 | 684911 | Zhang Xiaowen | 2 | 56 | L7 | 3 | inflammation |  | inflammation |  |  |
| 2 | 696012 | Qu Zhaojun | 1 | 48 | R7 | 3 | adenocarcinoma | 1 |  |  |  |
| 2 | 525362 | Jiang Shengli | 1 | 51 | R2 | 1 | inflammation |  |  | SCLC |  |
| 2 | 467360 | Liu Yingjie | 2 | 59 | R8 | 3 | Breast cancer metastasis |  |  |  |  |
| 2 | 698130 | Jiang Cuifeng | 2 | 77 | R1 | 1 | adenocarcinoma | 1 |  |  |  |
| 2 | 666044 | Kong Fanjie | 1 | 50 | R3 | 1 | inflammation |  |  |  | no conclision |
| 2 | 260983 | Tian Xinli | 1 | 70 | R6 | 3 | inflammation |  |  | SCLC |  |
| 2 | 666835 | Zhi Li | 2 | 35 | L6 | 3 | inflammation |  |  |  | pneumonia |
| 2 | 667391 | Zhang Yilan | 2 | 76 | R7 | 3 | inflammation | adenocarcinoma |  |  |  |
| 2 | 666890 | Song Shixiang | 1 | 67 | R10 | 3 | inflammation |  |  | adenocarcinoma |  |
| 2 | 673911 | Liu Yuefu | 1 | 71 | L3 | 1 | inflammation |  |  |  | pneumonia |
| 2 | 676163 | Liu Shinian | 1 | 71 | R10 | 3 | inflammation |  |  | SCLC |  |
| 2 | 678177 | Tang Zhiguo | 1 | 65 | R6 | 3 | inflammation |  |  |  | no conclision |
| 2 | 392975 | He Qinglin | 1 | 64 | R3 | 1 | squamous carcinoma |  |  |  |  |
| 2 | 673100 | Luo Guolan | 2 | 74 | R6 | 3 | inflammation |  | inflammation |  |  |
| 2 | 391156 | Zhang Fengjun | 1 | 51 | R2 | 1 | inflammation |  |  |  | pneumonia |
| 2 | 682639 | Hou Guifang | 2 | 63 | L2 | 1 | adenocarcinoma | 1 |  |  |  |
| 2 | 619113 | Fu Shoukuan | 1 | 63 | R10 | 3 | inflammation |  |  |  | no conclision |

Note: group 1 is patients that EBUS-GS-TBCB is performed, group 2 is patients that EBUS-GS-TBB is performed. 1 in the line of gender stands for male, 2 in the line of gender stands for female. R in the line of position stands for right, L in the line of position stands for left, the number in the line of position stands for pulmonary areas affiliated of corresponding segmental bronchus. 1 in the line of distribution stands for superior lung area included apical, anterior and posterior pulmonary segments of left and right upper lobe, 2 in the line of distribution stands for middle lung area included the right middle lobe and lingual pulmonary segment of left upper lobe, 3 in the line of distribution stands for inferior lung area included left and right lower lobe. 1 in the line of operation stands for surgical pulmonary resection being performed and pathological diagnosis don’t change. TTCB: transthoracic needle aspiration or core biopsy. SCLC: small cell lung cancer.
