# Supplementary material for: Diagnosis of Peripheral Pulmonary Lesions with Transbronchial Lung Cryobiopsy by Guide Sheath and Radial Endobronchial Ultrasonography: A Prospective Control Study
Source: Can Respir J. 2021 Sep 28;2021:6947037. doi: 10.1155/2021/6947037 (PMC8492292; doi:10.1155/2021/6947037)
Supplement: Supplementary Materials — The clinical information and final diagnostic tests groups EBUS-GS-TBCB and EBUS-GS-TBB are shown in Supplement Table 1. The final diagnosis, the shortest pleural distance, orientation of radial image, and complication in groups EBUS-GS-TBCB and EBUS-GS-TBB are shown in Supplement Table 2. [file 6947037.f1.zip › 6947037.f1/supplement 2(2) (2).doc]

Supplement Table 2 the final diagnosis, pleural distance, orientation of radial image and complication in two groups

| Grouping | Case number | Name | Gender | Age | Final diagnosis | Pleural distance | Orientation of radial-EBUS | Pneumothorax | Bleeding | diameter |
| --- | --- | --- | --- | --- | --- | --- | --- | --- | --- | --- |
| 1 | 655793 | Shi Enhai | 1 | 79 | Sarcomatoid carcinoma | 27.74 | C | . | . | 30.78 |
| 1 | 657456 | Qu Jianguo | 1 | 65 | adenocarcinoma | 32.34 | C |  | 2 | 53.83 |
| 1 | 659717 | Chen Anzuo | 1 | 50 | squamous carcinoma | 20.43 | C | . | . | 33.27 |
| 1 | 661361 | Hou Jiaqi | 1 | 68 | adenocarcinoma | 16.55 | C | . | . | 28.61 |
| 1 | 388006 | Mu Jianguo | 1 | 63 | adenocarcinoma | 36.84 | C | 2 | 1 | 43.32 |
| 1 | 680612 | Dong Zhenfu | 1 | 66 | adenocarcinoma | 45.65 | C | . | . | 32.28 |
| 1 | 680175 | Zhang Tao | 1 | 53 | adenocarcinoma | 29.45 | C | . | . | 26.85 |
| 1 | 681610 | Chi Yunping | 2 | 60 | granuloma TB | 9.21 | C | . | 1 | 26.77 |
| 1 | 693890 | Sun Qingrong | 2 | 74 | adenocarcinoma | 17.66 | C | . | . | 28.23 |
| 1 | 157386 | Wu Yanfeng | 2 | 64 | granuloma TB | 8.10 | C | 1 | . | 15.89 |
| 1 | 681468 | Li Wenlai | 1 | 61 | SCLC | 7.46 | C | . | . | 19.77 |
| 1 | 658457 | Liu Guoliang | 1 | 65 | Kidney cancer metastasis | 35.66 | E | . | . | 48.53 |
| 1 | 669540 | Wang Xiaoyuan | 2 | 51 | adenocarcinoma | 15.20 | E | . |  | 41.33 |
| 1 | 671001 | Shao Gonghe | 1 | 74 | adenocarcinoma | 8.61 | E | . | . | 30.78 |
| 1 | 673289 | Hou Liping | 2 | 66 | adenocarcinoma | 20.06 | C | . | . | 23.34 |
| 1 | 698751 | Wang Peiyuan | 1 | 45 | adenocarcinoma | 25.44 | C | . | . | 12.00 |
| 1 | 698749 | Cui Weiqing | 1 | 66 | adenocarcinoma | 30.02 | C | . | 1 | 41.01 |
| 1 | 180188 | Yang Xiyou | 1 | 66 | adenocarcinoma | 14.70 | C | . | . | 51.16 |
| 1 | 600642 | Jiang Dejun | 1 | 72 | inflammation | 26.65 | C | . | 2 | 41.12 |
| 1 | 699401 | Shi Shufa | 1 | 72 | adenocarcinoma | 24.33 | C | . | . | 20.55 |
| 1 | 699927 | Wang Mingchun | 1 | 74 | adenocarcinoma | 12.75 | C | . | . | 16.93 |
| 1 | 554767 | Zhang Zongxian | 1 | 67 | adenocarcinoma | 65.55 | C | . | . | 24.66 |
| 1 | 187362 | Liu Huanyu | 2 | 69 | breast cancer metastasis | 45.95 | C | . | . | 14.12 |
| 1 | 627563 | Yu Guilan | 2 | 68 | adenocarcinoma | 31.71 | C | . | . | 24.67 |
| 1 | 704242 | Wang Hongwei | 2 | 65 | No conclusion | 19.00 | C | . | . | 33.00 |
| 1 | 705628 | Han Min | 2 | 61 | adenocarcinoma | 13.92 | C | . | 1 | 27.06 |
| 1 | 705489 | Yao Shuhua | 2 | 62 | hylactic pneumonia | 11.78 | C | . | . | 35.77 |
| 1 | 566434 | Li Xueming | 1 | 60 | adenocarcinoma | 13.33 | C | . | . | 20.89 |
| 2 | 643684 | Sun Maosong | 1 | 56 | SCLC | 37.36 | C | . | . | 33.68 |
| 2 | 651563 | Gao Shenglin | 1 | 74 | adenocarcinoma | 18.81 | C | . | . | 44.24 |
| 2 | 653893 | Wen Guichun | 2 | 64 | adenocarcinoma | 9.74 | C | . | . | 26.64 |
| 2 | 653638 | Zhang Wei | 1 | 61 | adenocarcinoma | 20.98 | E | . | . | 35.77 |
| 2 | 656123 | Zhang Chunyun | 2 | 66 | adenocarcinoma | 22.49 | C | . | . | 37.66 |
| 2 | 656657 | Jia Xuemei | 2 | 50 | no conclusion | 15.35 | C | . | . | 45.22 |
| 2 | 658493 | Jiang Chengshuang | 1 | 63 | adenocarcinoma | 17.26 | C | . | . | 32.11 |
| 2 | 658516 | Zhang Yicheng | 1 | 55 | SCLC | 13.60 | C | . | . | 23.32 |
| 2 | 663861 | Wang Xiaoxiao | 2 | 32 | adenocarcinoma | 20.38 | C | . | . | 11.63 |
| 2 | 666416 | Li Zhigeng | 1 | 67 | squamous carcinoma | 18.61 | C | . | . | 30.90 |
| 2 | 676813 | Shi Baosheng | 1 | 79 | adenocarcinoma | 18.34 | E | . | . | 45.70 |
| 2 | 679360 | Lu Mengjing | 1 | 60 | adenocarcinoma | 23.54 | C | . | . | 24.30 |
| 2 | 684333 | Wang Baozhen | 1 | 79 | adenocarcinoma | 16.94 | E | . | . | 16.35 |
| 2 | 684911 | Zhang Xiaowen | 2 | 56 | inflammation | 17.10 | C | . | . | 30.32 |
| 2 | 696012 | Qu Zhaojun | 1 | 48 | adenocarcinoma | 18.47 | C | . | 1 | 25.12 |
| 2 | 525362 | Jiang Shengli | 1 | 51 | SCLC | 17.14 | C | . | . | 25.33 |
| 2 | 467360 | Liu Yingjie | 2 | 59 | breast cancer metastasis | 13.26 | C | . | . | 34.21 |
| 2 | 698130 | Jiang Cuifeng | 2 | 77 | adenocarcinoma | 36.33 | C | . | . | 33.65 |
| 2 | 666044 | Kong Fanjie | 1 | 50 | no conclusion | 13.81 | E | . | . | 21.66 |
| 2 | 260983 | Tian Xinli | 1 | 70 | SCLC | 8.41 | E | . | . | 24.30 |
| 2 | 666835 | Zhi Li | 2 | 35 | pneumonia | 36.87 | E | . | . | 16.49 |
| 2 | 667391 | Zhang Yilan | 2 | 76 | adenocarcinoma | 17.66 | C | . | . | 27.46 |
| 2 | 666890 | Song Shixiang | 1 | 67 | adenocarcinoma | 18.66 | C | . | . | 19.13 |
| 2 | 673911 | Liu Yuefu | 1 | 71 | pneumonia | 15.15 | C | . | . | 19.45 |
| 2 | 676163 | Liu Shinian | 1 | 71 | SCLC | 30.03 | C | . | 1 | 20.03 |
| 2 | 678177 | Tang Zhiguo | 1 | 65 | no conclusion | 20.51 | C | . | . | 26.35 |
| 2 | 392975 | He Qinglin | 1 | 64 | squamous carcinoma | 42.32 | C | . | . | 26.73 |
| 2 | 673100 | Luo Guilan | 2 | 74 | inflammation | 23.22 | C | . | . | 25.77 |
| 2 | 391156 | Zhang Fengjun | 1 | 51 | pneumonia | 21.30 | C | . | . | 32.55 |
| 2 | 682639 | Hou Guifang | 2 | 63 | adenocarcinoma | 9.54 | C | . | . | 41.33 |
| 2 | 619113 | Fu Shoukuan | 1 | 63 | no conclusion | 15.55 | C | . | . | 31.92 |

Note: group 1 is patients that EBUS-GS-TBCB is performed, group 2 is patients that EBUS-GS-TBB is performed. 1 in the line of gender stands for male, 2 in the line of gender stands for female. C in the line of orientation of radial-EBUS stands for concentric images that hypoechoic area centre round radial probe, E in the line of orientation of radial-EBUS stands for eccentric images that hypoechoic areas revolve a side of radial probe. 1 in the line of pneumothorax stands for light degree of pneumothorax, 2 in the line of pneumothorax stands for severe pneumothorax. 1 in the line of bleeding stands for light degree of bleeding, 2 in the line of bleeding stands for severe bleeding.
